# Single-cell expression profiles of *ACE2* and *TMPRSS2* reveals potential vertical transmission and fetus infection of SARS-CoV-2

**DOI:** 10.18632/aging.104015

**Published:** 2020-10-26

**Authors:** Mengdie Lü, Li Qiu, Guangshuai Jia, Rongqun Guo, Qibin Leng

**Affiliations:** 1 Affiliated Cancer Hospital and Institute of Guangzhou Medical University, State Key Laboratory of Respiratory Disease, Guangzhou, China; 2 Joint National Laboratory for Antibody Drug Engineering, Key Laboratory of Cellular and Molecular Immunology of Henan Province, Institute of Translational Medicine, School of Basic Medicine, Henan University, Kaifeng, China; 3 Department of Clinical Oncology, Taihe Hospital, Hubei University of Medicine, Shiyan, Hubei, China; 4 Department of Hematology, The First Affiliated Hospital of Zhengzhou University, Zhengzhou, Henan, China

**Keywords:** COVID-19, SARS-CoV-2, pathogenesis, fetus, placenta

## Abstract

Morbidity and mortality of coronavirus disease 2019 (COVID-19) is age-dependent. It remains unclear whether vertical severe acute respiratory syndrome coronavirus 2 (SARS-CoV-2) occurs during pregnancy and how such infection will affect fetal development. Here, we performed single-cell transcriptomic analysis of placenta and other tissues from fetuses in comparison with those from adults using public-available datasets. Our analysis revealed that a very small proportion of trophoblast cells expressed the *Angiotensin I Converting Enzyme 2* (*ACE2*) gene, suggesting a low possibility of vertical transmission of SARS-CoV-2 from mother to fetus during pregnancy. We found that the fetal adrenal gland, heart, kidney and stomach were susceptible to SARS-CoV-2 infection, because these organs contained cell clusters that expressed high levels of the *ACE2* gene. In particular, a higher proportion of *ACE2*-expressing cell clusters in the adrenal gland and kidney also expressed the *Transmembrane Serine Protease 2* (*TMPRSS2*) gene compared with other organs. Surprisingly, *ACE2*-expressing type II alveolar (AT2) equivalent cells were absent in fetal lungs. This is in sharp contrast to adult lungs. As *ACE2* expression is regulated by various conditions, including oxygen concentration, inflammation and smoking, caution is warranted to avoid triggering potential *ACE2* expression in fetal and placental tissue.

## INTRODUCTION

At present, the worldwide pandemic of SARS-CoV-2 virus have caused serious health, economic and social problems. It is reported that the potential key factors for mortality are an older age and chronic comorbidities [1]. Fever and cough are the most common symptoms, followed by sputum production and fatigue [2]. Consistent with the symptoms, autopsy findings in adults included damage in the lungs, as well as the adrenal gland, blood vessels, gallbladder, heart, kidney and liver [3, 4]. Children are also susceptible to SARS-CoV-2 infection, but have relatively mild symptoms [5]. Recently, a serological SARS-CoV-positive and PCR-negative newborn case was reported [6], suggesting possible vertical transmission of SARS-CoV-2 during pregnancy. Thus, the risk of vertical transmission and the potential pathogenesis of fetuses infected with SARS-CoV-2 merit further investigation.

Human angiotensin-converting enzyme 2 (ACE2) was reported as the docking and entry receptor of SARS-like coronavirus to invade human cells [7, 8]. The ACE2 was also identified as a cellular entry receptor for SARS-CoV-2, which did not use other coronavirus receptors, such as aminopeptidase N and dipeptidyl peptidase 4 [9]. Moreover, the priming of SARS-CoV-2 spike protein by cellular transmembrane serine protease 2 (TMPRSS2) was shown to be essential for viral entry [10]. Single-cell RNA sequencing (scRNA-seq) analysis has revealed that *ACE2* is expressed mainly on AT2 cells in the lungs [11]. The AT2 cells also express many other genes that are involved in viral replication and transmission [12]. Thus, AT2 cells represent the most susceptible cells for SARS-CoV-2 infection in adult lungs. In addition, *ACE2* is also expressed in bronchial and nasal epithelial cells, absorptive enterocytes from ileum and colon, liver cholangiocytes and cells from kidney proximal tubules [11, 13, 14]. These studies suggest that SARS-CoV-2 infection may directly contribute to the pathogenesis of respiratory and digestive system diseases in patients with severe COVID-19 infection.

To assess the risk of vertical infection of the fetus during pregnancy, we analyzed *ACE2* and *TMPRSS2* gene expression in human placenta and fetal tissues compared with adult tissues using publicly-available scRNA-seq datasets (GSE134355) [15]. Our analysis revealed a low level of potential infection of the placenta with SARS-CoV-2. In addition, fetal lungs appear unlikely to be susceptible to SARS-CoV-2 viral infection.

## RESULTS

### Few *ACE2* expressing trophoblast cells in the placenta indicate a low possibility of vertical transmission of SARS-CoV-2 infection

**Figure 1:**
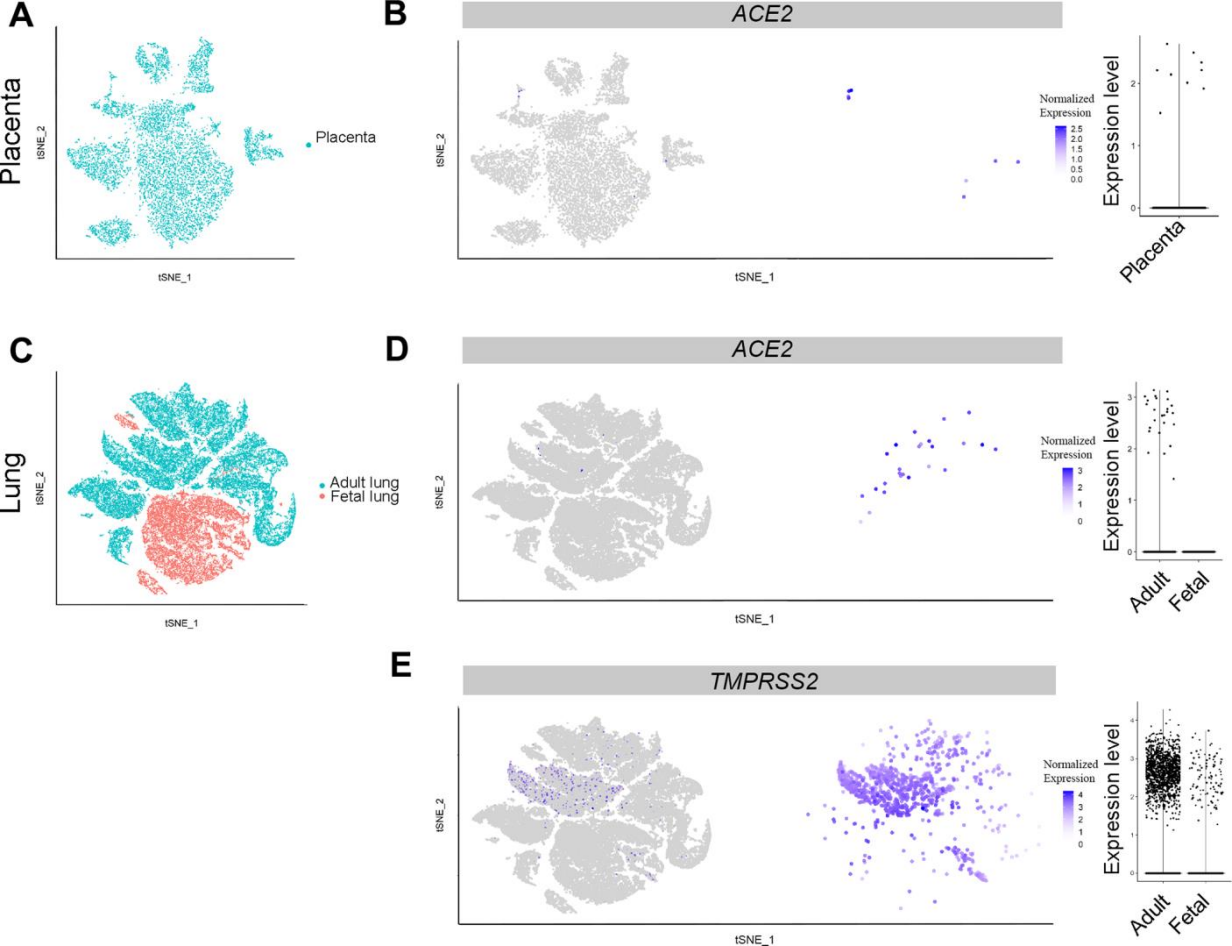
**Single-cell analysis of lungs and placenta.** (**A**) t-distributed stochastic neighbor embedding (TSNE) plot showing sub-clusters of placenta cells, and (**B**) *ACE2* expression in placenta. (**C**) TSNE plot showing sub-clusters of lung cells, (**D**) *ACE2* and (**E**) *TMPRSS2* expression in lungs from the adult and fetal groups.

The placenta is a transient organ critical for normal embryonic development [16]. To explore the possibility of vertical transmission of SARS-CoV-2 infection during pregnancy, we analyzed single-cell data from the placenta. Nine *ACE2*-positive cells, which were located in the *GATA3*-positive population, were detected among 9,852 placenta-derived cells (Figure 1A, 1B). Moreover, the *GATA3*-positive cell clusters were also enriched for *CSH1*, *KRT19*, and *COL1A1* expression, markers for trophoblast cells [16–18] (Supplementary Figure 1A). This evidence indicates that SARS-CoV-2 may directly infect trophoblast cells. For example, a recent case report found transplacental transmission of SARS-CoV-2 infection, identified by SARS-CoV-2 positive trophoblastic cells in the placenta [19]. Of note, placental barrier trophoblast cells also secrete interferon-λ1 to protect the fetus from viral infection [20]. Thus, any conditions that increase ACE2-expressing trophoblasts or diminish their ability to secret interferon-λ1 will potentially enhance the possibility of vertical infection of SARS-CoV-2.

### Fetal lungs and liver are likely not susceptible to SARS-CoV-2 infection

**Figure 2:**
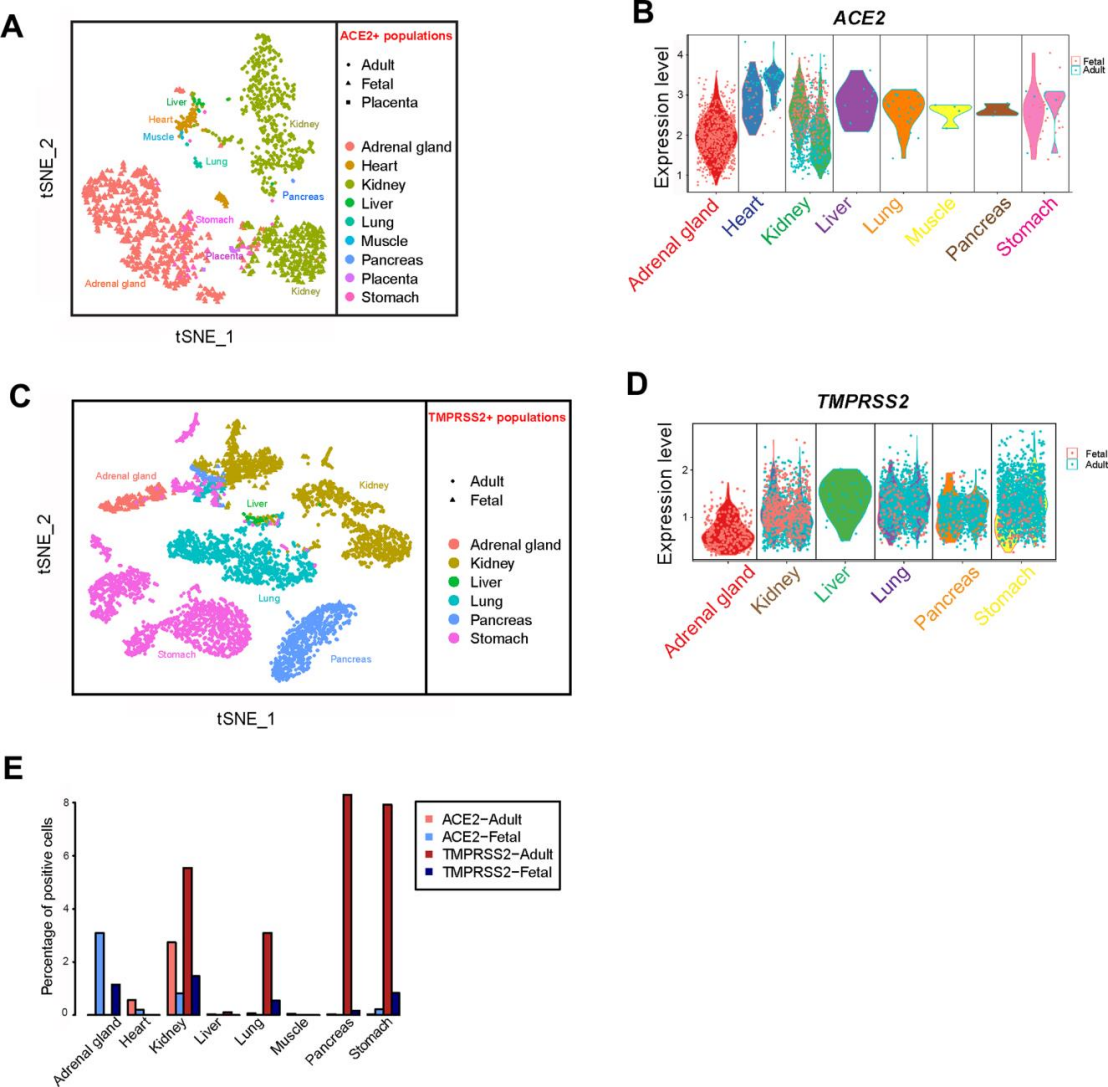
**Profiling of *ACE2*- and *TMPRSS2*-positive cells in different tissues.** (**A**) t-distributed stochastic neighbor embedding (TSNE) plot of *ACE2*-positive cells. Cells from the merged adult, fetal and placenta groups are indicated by different dot shapes. (**B**) Violin plot of *ACE2* expression in different tissues from the adult and fetal groups. (**C**) TSNE plot of *TMPRSS2*-positive cells. Cells from the merged adult, fetal and placenta groups are indicated by different dot shapes. (**D**) Violin plot of *TMPRSS2* expression in different tissues from the adult and fetal groups. (**E**) Bar plot showing the percentage of cells expressing *ACE2* and *TMPRSS2* in the adult and fetal groups.

Lungs are a primary target organ for SARS-CoV-2 infection. In addition, severe pneumonia is the major reason for fatality of patients with COVID-19 infection [1, 14]. To better understand the potential pathogenesis of COVID-19 infection in the fetus, we analyzed scRNA-seq data from the GEO database (GSE134355: lungs) (Table 1). A total of 57,011 cells from five samples were harvested after merge using the Seurat R package. After filtration, 56,726 cells qualified for further data analysis, with 18,951 from fetal lungs tissue. Cell clusters were identified using reported markers, namely *CAV1* and *AGER* (AT1 cells) [21], *SFTPC*, *ABCA3* (AT2 cells), *NKX2-1*, *FOXA2* [22], *PTPRC* (hematopoietic cells), *COL1A1* (fibroblast cells) and *CLDN5* (endothelial cells) [23] (Supplementary Figure 1B). Among the clusters, adult AT2 cells widely expressed the *TMPRSS2* gene compared with the *ACE2* gene. In contrast, equivalent AT2 cells in the fetus did not express *TMPRSS2*. Noticeably, a small fraction of progenitor cells (*NKX2-1*+) in fetal lungs expressed *TMPRSS2* (Figure 1C, 1E, Supplementary Figure 1B). To our surprise, *ACE2*-positive cells were absent in the fetus, whereas 0.66% of adult AT2 cells expressed the *ACE2* gene (Figure 1C, 1D, Figure 2). Furthermore, TMPRSS2 alone without ACE2 will not mediate SARS-CoV-2 infection efficiently. Therefore, these results suggest that fetal lungs are unlikely to be susceptible to SARS-CoV-2 infection, because of the absence of *ACE2*-expressing cells.

**Table 1:** Description of sample information used in this study.

**Tissue**	**GSM_ID**	**Type**	**Age**	**Sex**
Lung	GSM4008628	Adult	21y	M
Lung	GSM4008629	Adult	21y	F
Lung	GSM4008630	Adult	49y	F
Lung	GSM4008631	Adult	49y	F
Lung	GSM4008632	Adult	49y	F
Lung	GSM4008633	Adult	49y	F
Lung	GSM4008699	Fetal	12w	F
Lung	GSM4008700	Fetal	11w	F
Heart	GSM3980138	Adult	52y	M
Heart	GSM3980139	Adult	47y	F
Heart	GSM4008686	Fetal	12w	F
Heart	GSM4008687	Fetal	11w	F
Kidney	GSM4008619	Adult	66y	M
Kidney	GSM4008620	Adult	41y	M
Kidney	GSM4008621	Adult	57y	M
Kidney	GSM4008622	Adult	57y	M
Kidney	GSM4008693	Fetal	13w	M
Kidney	GSM4008694	Fetal	11w	M
Kidney	GSM4008695	Fetal	12w	M
Kidney	GSM4008696	Fetal	11w	F
Adrenal	GSM3943047	Adult	36y	M
Adrenal	GSM3943048	Adult	23y	F
Adrenal	GSM4008675	Fetal	12w	M
Adrenal	GSM4008676	Fetal	14w	M
Adrenal	GSM4008677	Fetal	12w	M
Adrenal	GSM4008720	Neonatal	6d	F
Adrenal	GSM4008721	Neonatal	6d	F
Liver	GSM4008623	Adult	21y	F
Liver	GSM4008624	Adult	22y	F
Liver	GSM4008625	Adult	52y	M
Liver	GSM4008626	Adult	23y	F
Liver	GSM4008627	Adult	24y	F
Liver	GSM4008697	Fetal	26w	F
Liver	GSM4008698	Fetal	27w	F
Muscle	GSM4008634	Adult	63y	M
Muscle	GSM4008703	Fetal	12w	M
Stomach	GSM4008651	Adult	45y	M
Stomach	GSM4008652	Adult	59y	M
Stomach	GSM4008653	Adult	62y	M
Stomach	GSM4008654	Adult	62y	M
Stomach	GSM4008655	Adult	62y	M
Stomach	GSM4008712	Fetal	7w	F
Tissue	GSM_ID	Type	Age	Sex
Stomach	GSM4008713	Fetal	11w	F
Pancreas	GSM4008637	Adult	43y	F
Pancreas	GSM4008704	Fetal	26w	F
Pancreas	GSM4008705	Fetal	11w	F
Pancreas	GSM4008706	Fetal	11w	F
Placenta	GSM4008722	Placenta	10w	-

**Figure 3:**
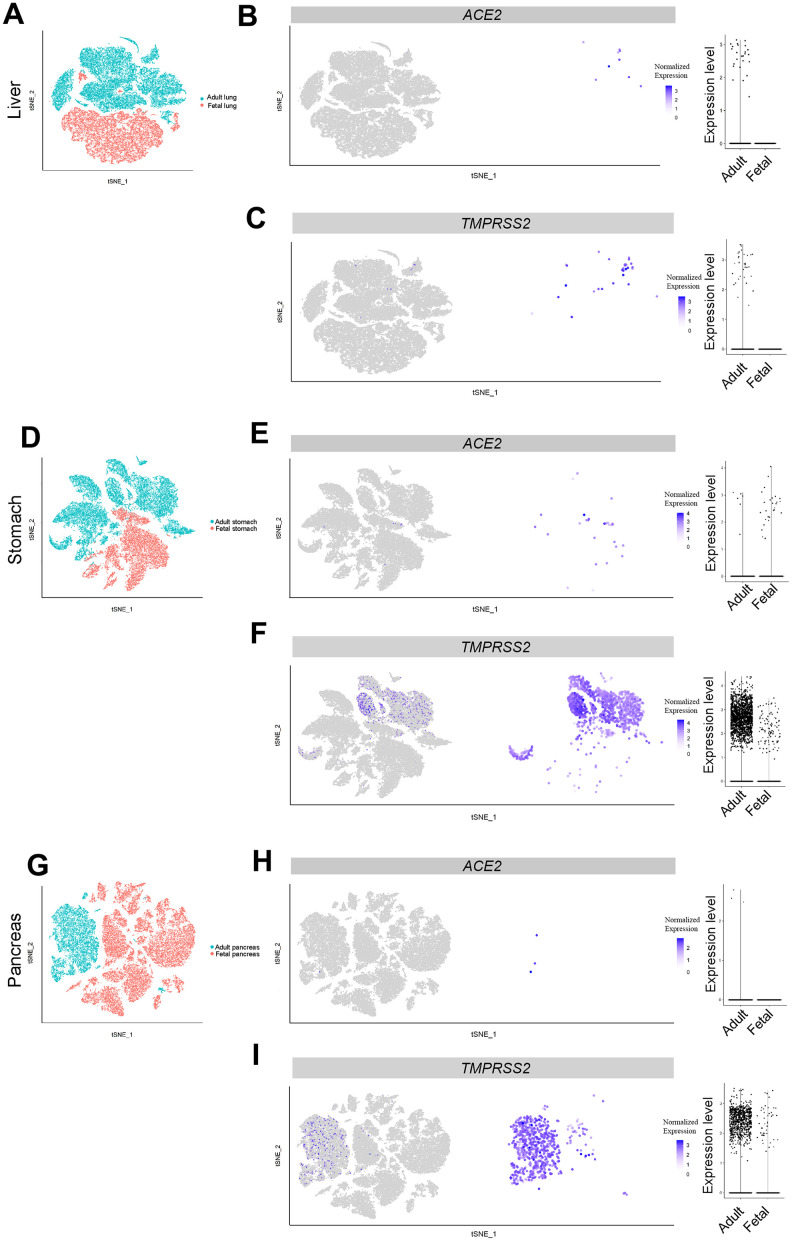
**Single-cell analysis of liver, pancreas and stomach.** (**A**) t-distributed stochastic neighbor embedding (TSNE) plot showing sub-clusters of liver cells, (**B**) *ACE2* and (**C**) *TMPRSS2* expression in liver from the adult and fetal groups. (**D**) TSNE plot showing sub-clusters of stomach cells, (**E**) *ACE2* and (**F**) *TMPRSS2* expression in stomach from the adult and fetal groups. (**G**) TSNE plot showing sub-clusters of pancreas cells, (**H**) *ACE2* and (**I**) *TMPRSS2* expression in pancreas from the adult and fetal groups.

To explore the susceptibility of the fetal liver to SARS-CoV-2 infection, we analyzed scRNA-seq data from five adult samples and two fetal samples. In total, 28,303 adult cells and 18,072 fetal cells were acquired after quality control screening. Although ten *ACE2*-expressing cells were detected in *CAV1*-positive clusters from the adult liver, all fetal liver cells were found to be *ACE2*-negative (Figure 3A, 3B, Supplementary Figure 1C). *TMPRSS2* was also exclusively detected in the *CAV1*-positive cluster of adult cells (Figure 3A, 3C, Supplementary Figure 1C). The expression pattern of *ACE2* and *TMPRSS2* genes in the *CAV1*-positive cluster was consistent with the finding that liver cholangiocytes were associated with liver injury in COVID-19 patients [24, 25]. Thus, our results suggest that adult liver but not fetal liver is susceptible to SARS-CoV-2 infection.

### Stomach but not pancreas is susceptible to SARS-CoV-2 infection

It was reported that *ACE2* is expressed in colonic epithelial cells and may be involved in SARS-CoV-2 infection [26]. To further investigate its expression pattern in the digestive system, we analyzed scRNA-seq datasets from the pancreas and stomach. In 22,479 adult stomach cells and 12,602 fetal stomach cells, a small fraction of cells expressed *ACE2* and *TMPRSS2* (Figure 3D–3F). The percentage of *ACE2*-positive stomach cells was higher in the fetus than in adult cells (27/12,602 vs 6/22,479 *ACE2*+, respectively) (Figure 2). These *ACE2*-expressing cells were mainly fibroblasts (*DCN-* and *VIM-*positive) and epithelium cells (*EPCAM-*positive) (Supplementary Figure 1D) [27]. In contrast, these *ACE2*-expressing cells were nearly absent in both the adult and fetal pancreas (Figure 3G, 3H). Expression of *TMPRSS2* was found in both pancreas and stomach tissues from adults and to a lesser extent from fetuses (Figure 3F, 3I). These data implied that adult and fetal stomach tissue is likely to be susceptible to SARS-CoV-2 viral infection. In comparison, the pancreas is less likely to be susceptible to infection.

### SARS-CoV-2 infection likely directly contributes to heart injury but may not directly impair muscle tissue

**Figure 4:**
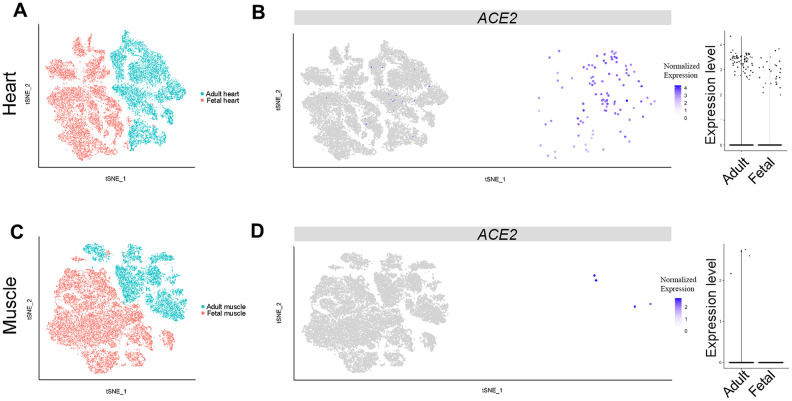
**Single-cell analysis of heart and muscle.** (**A**) t-distributed stochastic neighbor embedding (TSNE) plot showing sub-clusters of myocardial cells and (**B**) *ACE2* expression in myocardial cells from the adult and fetal groups. (**C**) TSNE plot showing sub-clusters of muscle cells and (**D**) *ACE2* expression in muscle from the adult and fetal groups.

Heart injury was highly correlated with poor prognosis of adult COVID-19 patients, probably because of direct virus infection through ACE2 [28, 29]. We analyzed the scRNA-seq dataset of adult and fetal heart tissues to identify different cell clusters (Supplementary Figure 1F). The percentage of *ACE2*-expressing cells was lower in fetal than in adult heart tissues (0.197% (29/14,713) vs 0.564% (68/12,063), respectively (Figure 4A, 4B, Figure 2). This result, together with previous reports [28, 29], suggested that SARS-CoV-2 may directly infect myocardial cells and contribute to heart injury in adults and to a less extent in fetuses.

We further analyzed scRNA-seq data of muscle tissues obtained from one adult and one fetus. The data included 9,582 and 19,787 qualified cells, respectively. The *ACE2*-expressing cells were found only in adult muscle cells and were absent in fetal tissue (Figure 4C, 4D). In addition, the proportion of *ACE2*-positive cells was very low (0.042%) compared with those of myocardial cells (Figure 2, Figure 4B), indicating muscle symptoms of COVID-19 patients may not directly result from SARS-CoV-2 infection.

### Cell clusters in fetal kidney and adrenal gland express both *ACE2* and *TMPRSS2* genes

**Figure 5:**
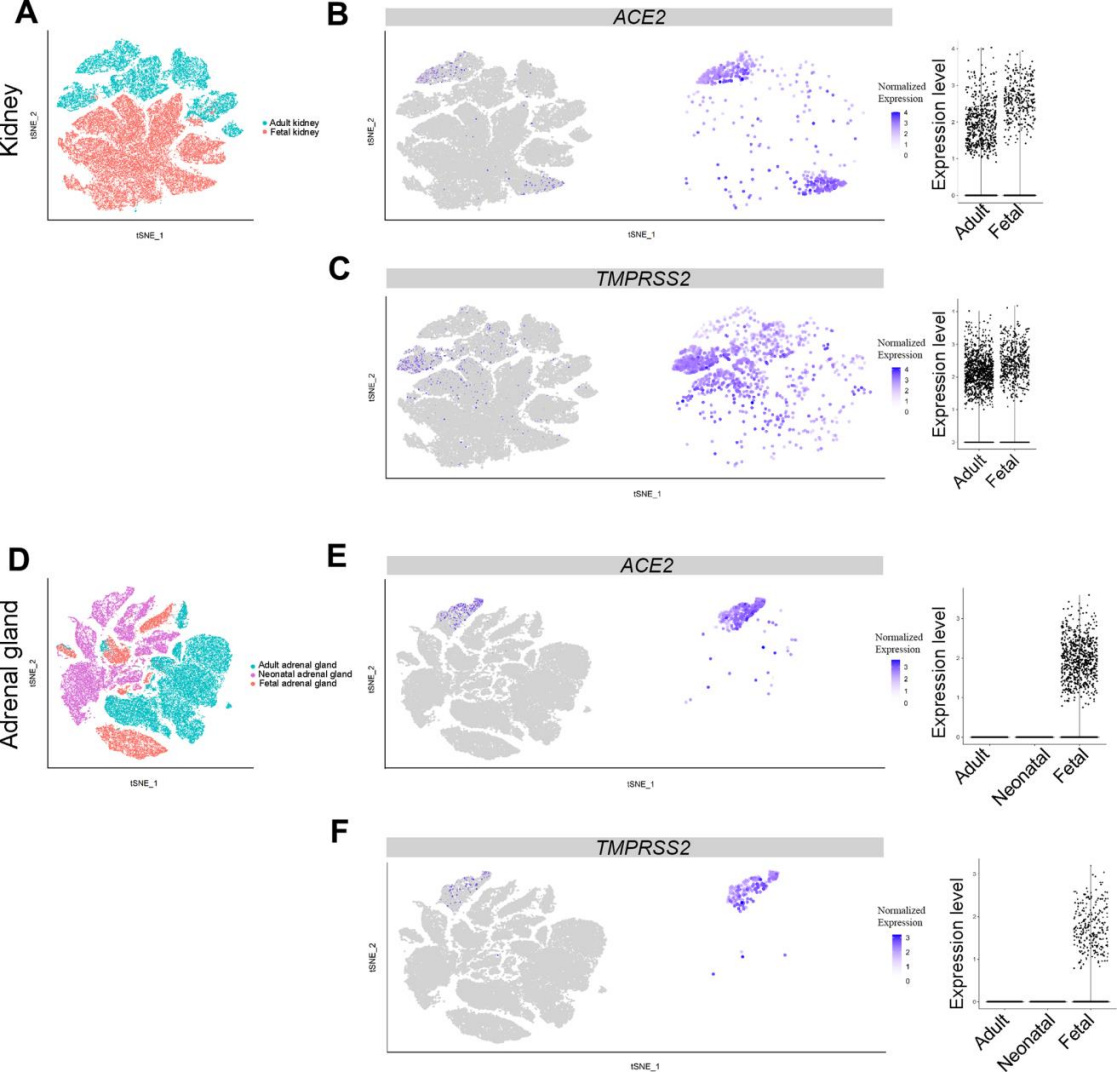
**Single-cell analysis of adrenal gland and kidney.** (**A**) t-distributed stochastic neighbor embedding (TSNE) plot showing sub-clusters of kidney cells, (**B**) *ACE2* and (**C**) *TMPRSS2* expression in kidney from the adult and fetal groups. (**D**) TSNE plot showing sub-clusters of adrenal gland cells, (**E**) *ACE2* and (**F**) *TMPRSS2* expression in adrenal gland from the adult, neonatal and fetal groups.

Acute renal injury is associated with the higher mortality rate of COVID patients [30]. Therefore, the kidney appears susceptible to SARS-CoV-2 infection. In total, 57,034 single cells from publicly-available data, consisting of three adult and four fetal samples, were analyzed for *ACE2* and *TMPRSS2* expression at the single-cell level. Expression of *PTPRC* was used as a marker for immune cells, *SLC34A1* for proximal tubules, *VCAM1* for parietal epithelial cells, *ATP6V0D2* for collecting duct intercalated cells and *SLC12A1* for cells of Henle’s loop (Supplementary Figure 1H) [31, 32]. The expression of *ACE2* was found mainly in proximal tubules of both adults and fetuses. The percentage of *ACE2*-expressing cells in the kidney was up to 2.736% among the 19,264 cells in the adult group, markedly higher than the other adult tissues. Fetal *ACE2* expression reached 0.818%, which was higher than the other fetal tissues (Figure 5A and 5B, Figure 2). The expression of *TMPRSS2* was mainly found in an *ATP6V0D2*-positive cluster in adults and widely expressed in fetal kidney cells (Figure 5A, 5C, Supplementary Figure 1H). These findings suggest that the kidney may be a main target for SARS-CoV-2 infection, beside the lungs, especially in the fetus.

**Figure 6:**
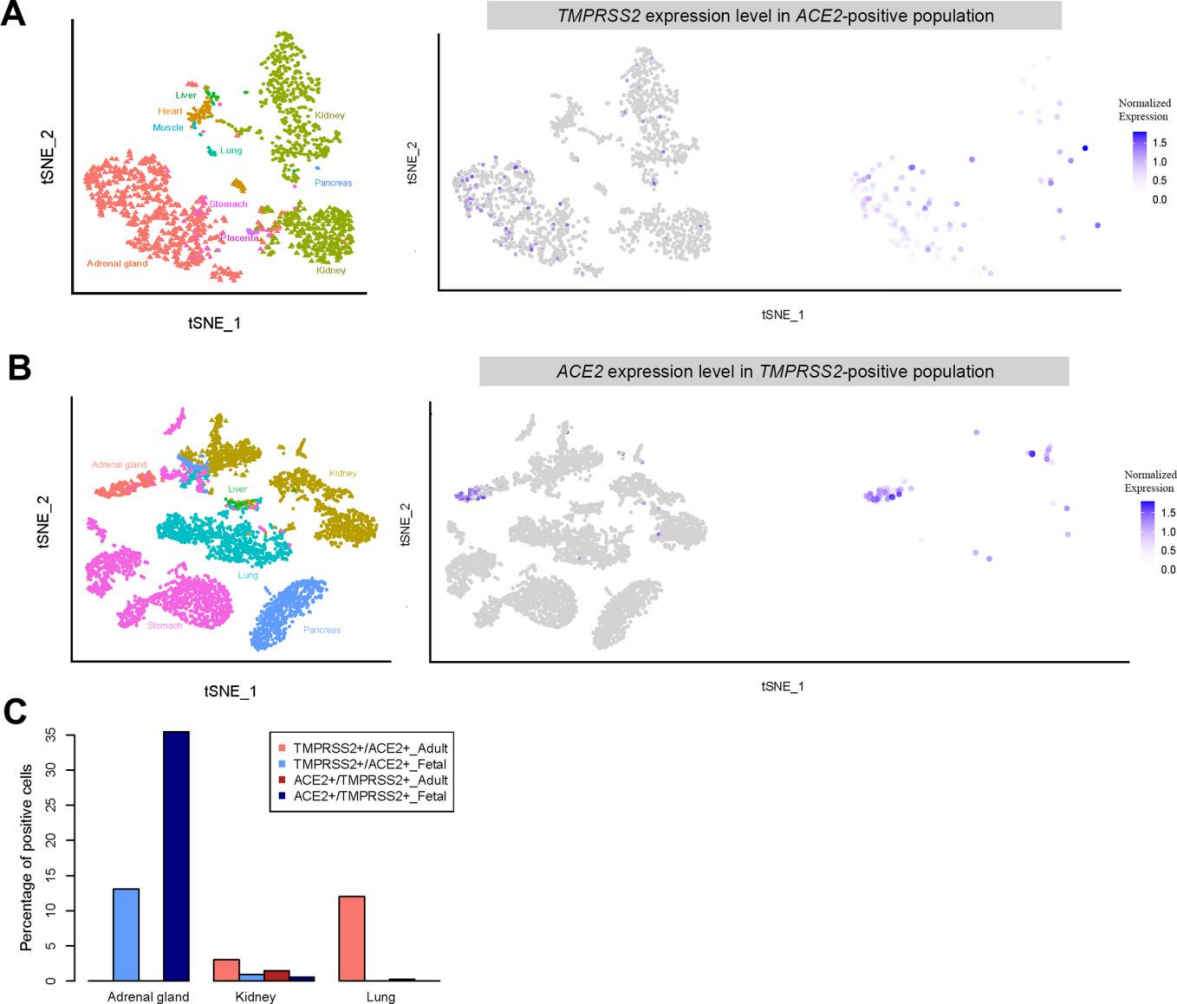
**Expression of double positive (*ACE2*- and *TMPRSS2*- positive) and single positive (*ACE2*- or *TMPRSS2*- positive) cells.** (**A**) t-distributed stochastic neighbor embedding (TSNE) plot displaying *TMPRSS2* expression in *ACE2*-positive cells. (**B**) TSNE plot showing *ACE2* expression in *TMPRSS2*-positive cells. (**C**) Percentage of double positive cells in each single positive population for the different tissues.

Further analysis revealed that a relatively high percentage of fetal adrenal gland cells expressed *ACE2* and *TMPRSS2* (694 and 257 cells were positive, respectively, in 22,498 total fetal single cells); 91 cells expressed both *ACE2* and *TMPRSS2* (Figure 6). Most of the *ACE2* and *TMPRSS2*-expressing cells were located in the *ELF3*+ cluster, which represents adrenal chromaffin cell precursors (Figure 5E, 5F, Supplementary Figure 1I) [33]. Furthermore, both *ACE2* and *TMPRSS2*-expressing cells were absent in the adrenal gland cells from either adults or neonates (Figure 5E, 5F). Thus, SARS-CoV-2 likely infects the fetal adrenal gland, but not the gland in neonates and adults.

## DISCUSSION

To evaluate the risk of vertical transmission and potential pathogenesis of SARS-CoV-2 infection in fetuses, we performed comparative single-cell analysis of *ACE2* and *TMPRSS2* expression in various adult and fetal tissues. We found that a few trophoblast cells expressed *ACE2* in the placenta. The proportion of these cells was very low compared to other tissues, suggesting that the possibility of vertical transmission may be also very low. Further analysis of additional datasets also confirmed the low expression level of *ACE2* in placenta and other fetal organs (Supplementary Figure 2). However, SARS-CoV-2 infection in newborn infants [6, 34, 35] highlights that some unknow factors may enhance the possibility of vertical transmission. Thus, our study encourages further investigation of the mechanisms underlying *ACE2* expression in trophoblast cells.

Consistent with previous studies [11, 12], we found *ACE2*-expressing cells were abundant in adult lungs AT2 cells. Surprisingly, *ACE2*-expressing cells were absent in fetal lungs, presumably resulting from the paucity of mature AT2 cells. Similarly, fetal liver lacked *ACE2*-expression in *CAV1*-positive clusters, which were present in adult liver. These results implied that fetal liver and lung tissues are not likely susceptible to SARS-CoV-2 infection.

Considering these findings, it is paradoxical that three out of 33 neonates were infected with SARS-CoV-2 when born to mothers with COVID-19. In particular, all three infected neonates had respiratory symptoms. One possibility is that lung *ACE2*-expression is higher in the peri-natal period of development. Our study examined scRNA-seq data from fetal tissues collected from 7-27 weeks of gestation. In addition, *ACE2* expression may be affected by oxygen concentration and other conditions, including smoking and inflammation [36]. Fetuses may not be harmed by the later environmental factors, perhaps explaining why *ACE2* expression was absent in their AT2 equivalent cells.

Similar to that in adults, muscle in fetuses appears unlikely to be susceptible to SARS-CoV-2 infection because of the lack of *ACE2*-expressing cells. Additionally, heart, kidney and stomach tissue from adults and fetuses all contained *ACE2*-expressing cells, suggesting SARS-CoV-2 infection may directly cause diarrhea and heart injury. Strikingly, a higher percentage of cells in the fetal adrenal gland and kidney expressed higher levels of both *ACE2* and *TMPRSS2* genes than those of adults. Thus, if vertical SARS-CoV-2 infection occurs during pregnancy, infection most likely will cause injury to both the adrenal gland and kidney. Because the adrenal gland secretes several important hormones, the infection may harm fetal growth and development.

## MATERIALS AND METHODS

### scRNA-seq datasets

All the scRNA-seq datasets from adult and fetal tissues were acquired from the GEO database (Accession NO. GSE134355) [15]. Fetal tissues included two lungs, two heart, one muscle, two liver, four kidney, three adrenal gland, two stomach and three pancreas tissue samples. The neonatal samples included two adrenal gland tissues. The adult tissues included one placenta, three lungs, two heart, one muscle, five liver, three kidney, two adrenal gland, one pancreas and three stomach tissue samples. The GSM numbers of all these samples are listed in Table 1.

### Quality control

Single-cell expression matrices were integrated for each tissue using “merge” function in the Seurat v3.1 R package [37]. Cells from lungs, liver and muscle tissues were filtered with a gene expression number per cell between 20 to 2500, and a mitochondrial gene expression percent below 30. Myocardial cells were filtered with a gene expression number per cell between 20 to 1000, and a mitochondrial percent below 30. Cells from adrenal gland and stomach tissues were filtered with a gene expression number per cell between 20 to 2500, and a mitochondrial percent below 40. Cells from pancreas and placenta tissues were filtered with a gene expression number per cell between 20 to 2500, and a mitochondrial percent below 20. Cells from kidney were filtered with a gene expression number per cell between 20 to 2500, with a mitochondrial percent below 50.

### Data processing

The merged datasets were scaled by the “ScaleData” function with regression of the variation of mitochondrial genes. The expression matrix was then normalized using the “NormalizeData” function. The top 5,000 variable genes were selected with the “FindVariableFeatures” function in the Seurat package [37]. To overcome the extensive technical noise in any single feature for scRNA-seq data, principal components were chosen by running the “JackStrawPlot” function followed by the visualization function “ElbowPlot”. Then, dimension reduction analysis was performed by the “RunPCA” function. The k-nearest neighbor graph based on the Euclidean distance was constructed using the “FindNeighbors” parameter, and cells were clustered by “FindClusters” with a ranged resolution from 0.3 to 0.7. Gene expression visualization based on non-linear dimensional reduction was shown by “DimPlot” and “FeaturePlot” parameters, and violin plots were obtained by the “VlnPlot” function.

## Supplementary Material

Supplementary Figures

Supplementary Table 1
