# Supplementary material for: Single-cell expression profiles of ACE2 and TMPRSS2 reveals potential vertical transmission and fetus infection of SARS-CoV-2
Source: Aging (Albany NY). 2020 Oct 26;12(20):19880–97. doi: 10.18632/aging.104015 (PMC7655214; doi:10.18632/aging.104015)
Supplement: Supplementary Figures [file aging-12-104015-s001..pdf]

SUPPLEMENTARY FIGURES

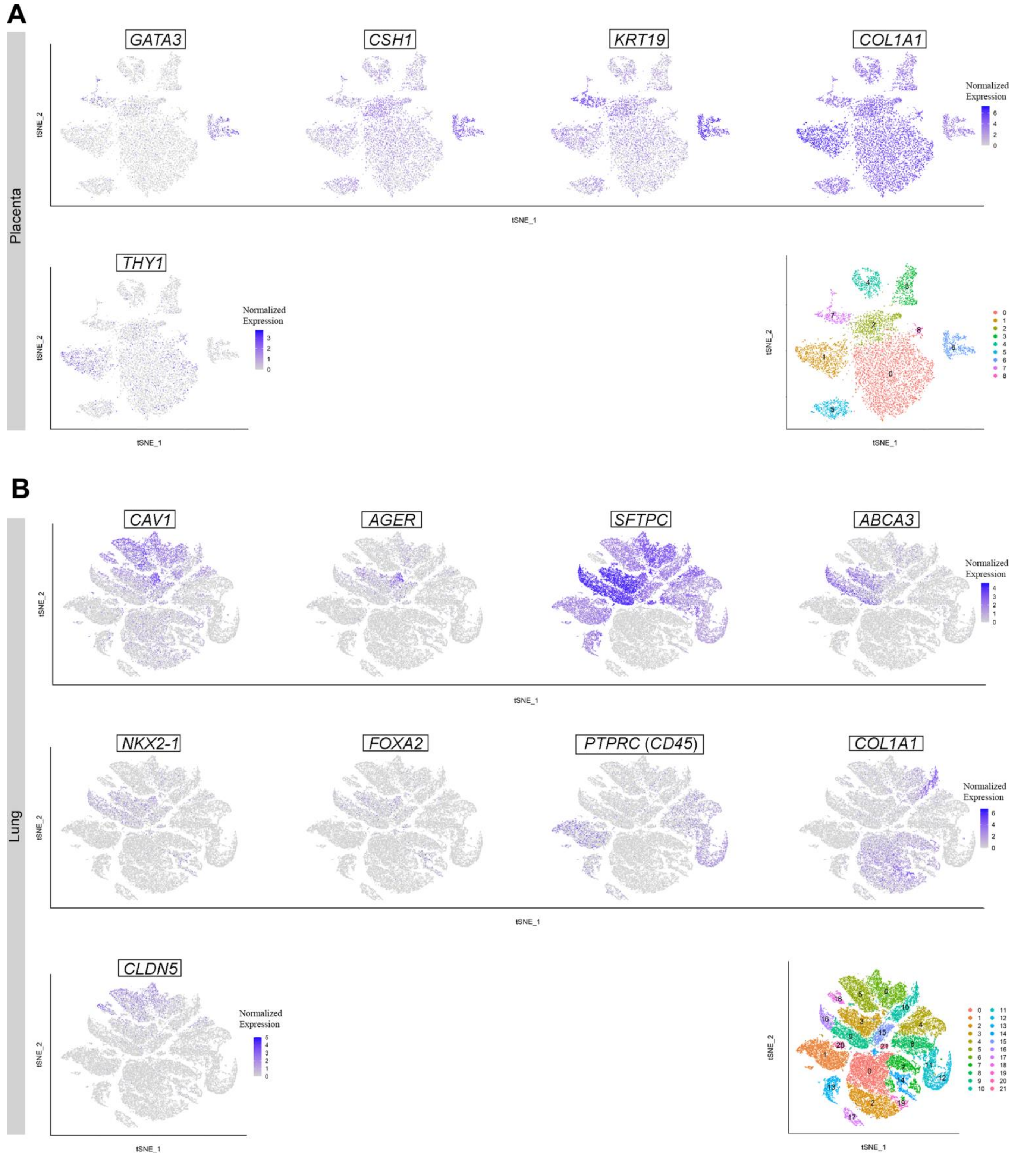

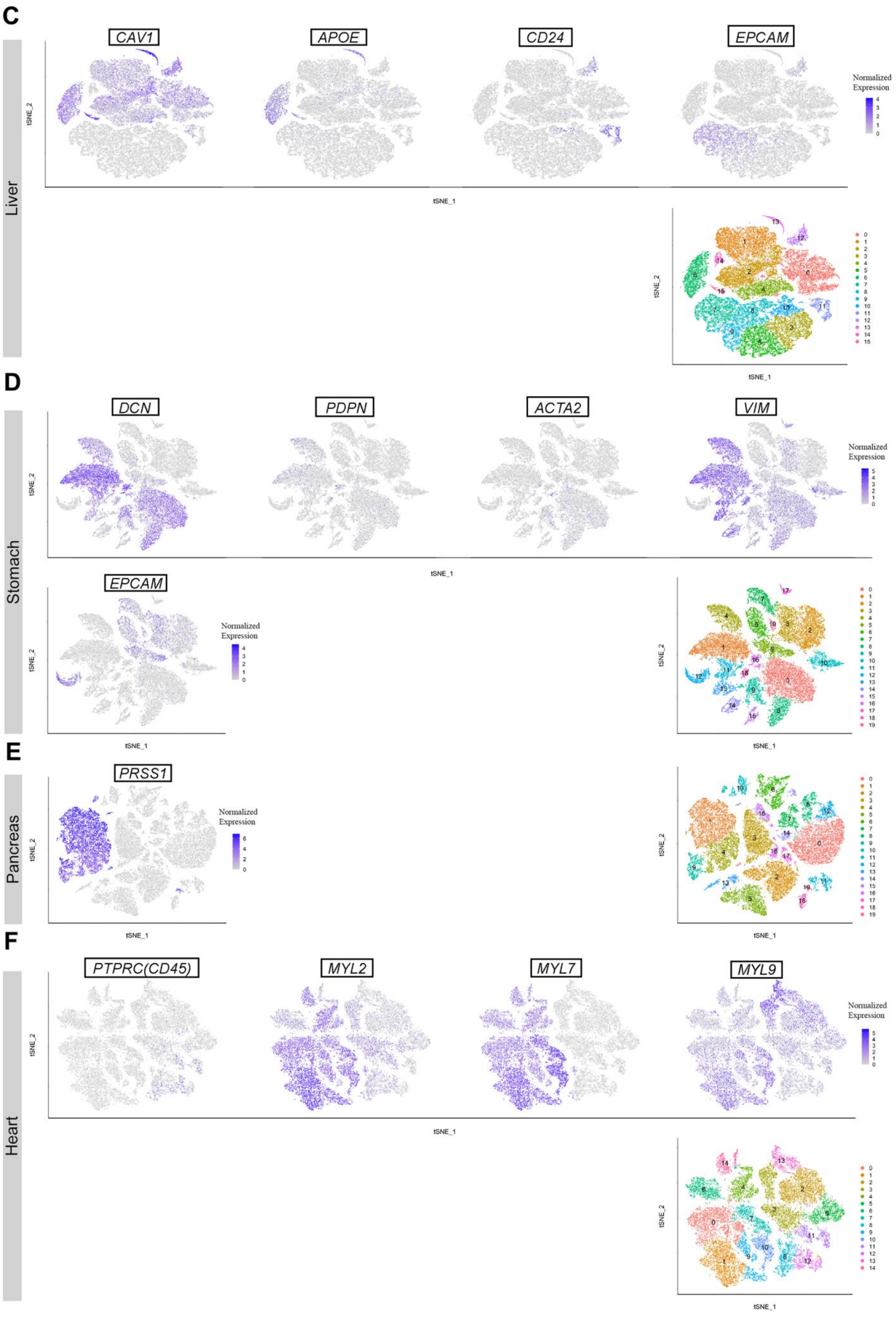
C
Liver
CAV1
APOE
CD24
EPCAM
Normalized Expression
tSNE_1
tSNE_2
D
Stomach
DCN
PDPN
ACTA2
VIM
EPCAM
E
Pancreas
PRSS1
F
Heart
PTPRC(CD45)
MYL2
MYL7
MYL9

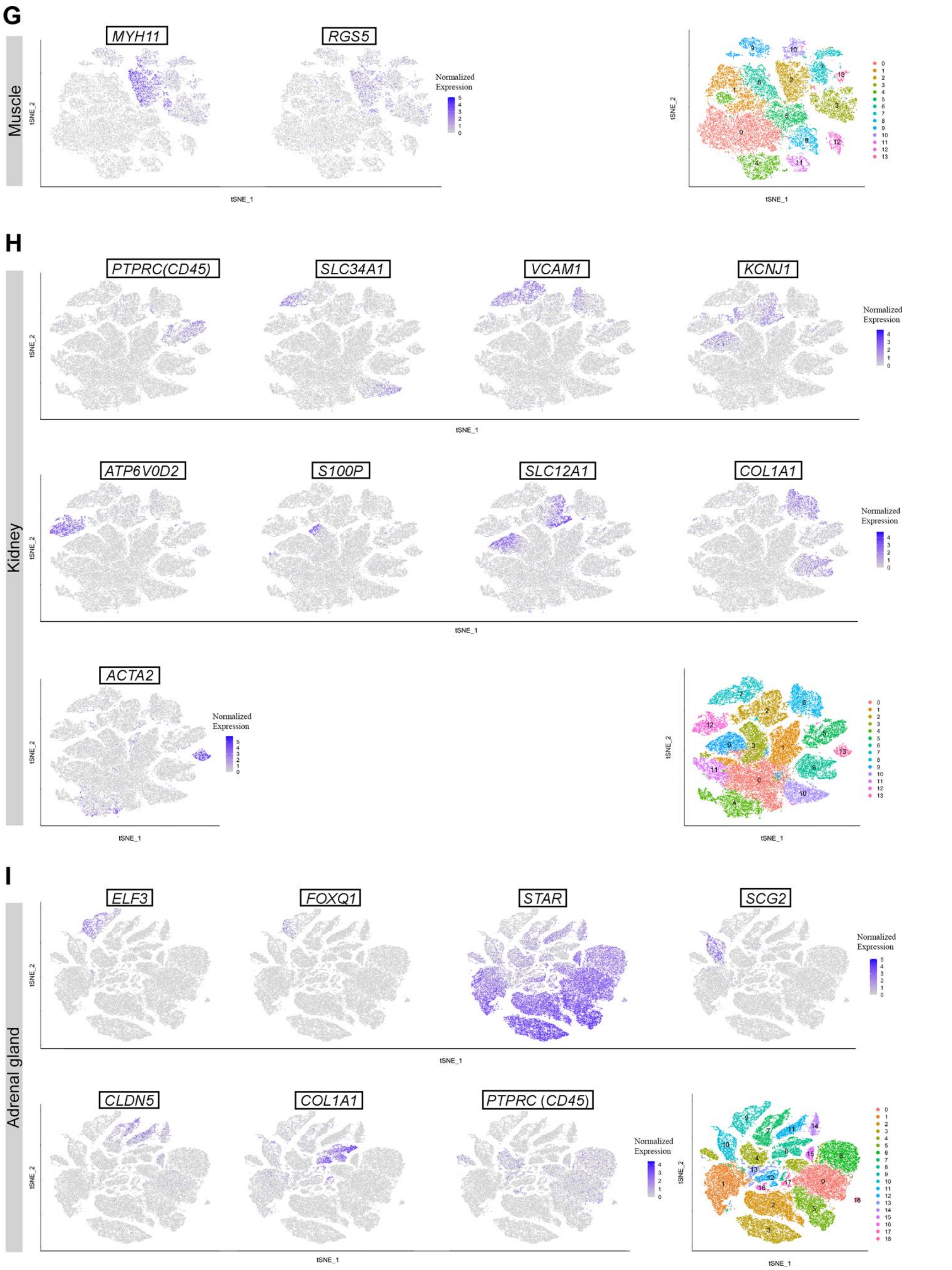

Supplementary Figure 1. Single-cell markers of each tissue. t-distributed stochastic neighbor embedding plot displaying cell markers of (**A**) placenta, (**B**) lungs, (**C**) liver, (**D**) stomach, (**E**) pancreas, (**F**) heart, (**G**) muscle, (**H**) kidney and (**I**) adrenal gland tissues.

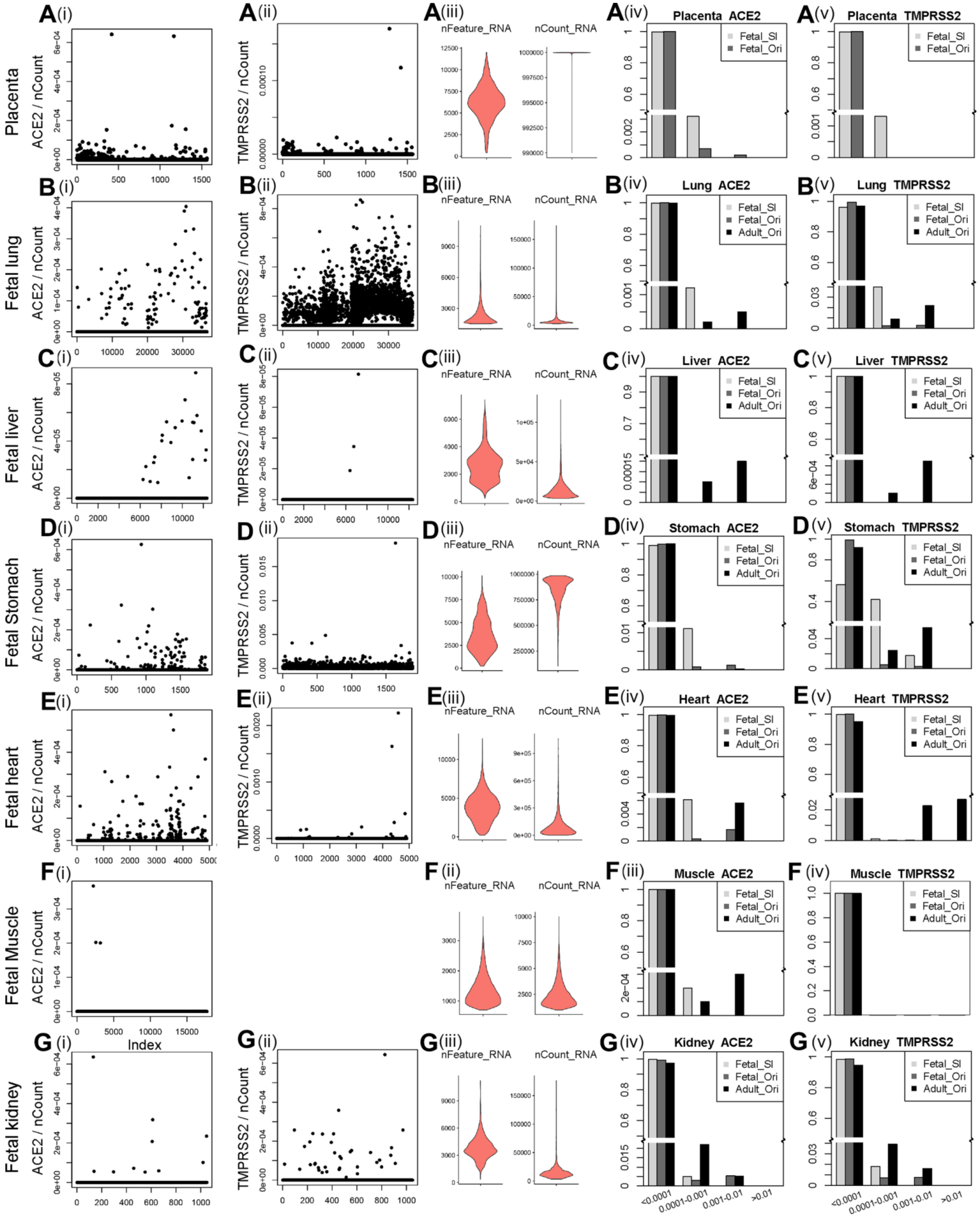

Supplementary Figure 2. *ACE2* and *TMPRSS2* expression levels in fetal tissues. **A** (i), **B** (i), **C** (i), **D** (i), **E** (i), **F** (i) and **G** (i) show the proportion of *ACE2* read counts versus total read counts in placenta, and fetal lungs, liver, stomach, heart, muscle and kidney, respectively. **A**

(ii), **B** (ii), **C** (ii), **D** (ii), **E** (ii), and **G** (ii) show the proportion of *TMPRSS2* read counts versus total read counts in placenta, and fetal lungs, liver, stomach, heart, muscle and kidney. **A** (iii), **B** (iii), **C** (iii), **D** (iii), **E** (iii), **F** (ii) and **G** (iii) show gene features and RNA counts detected in single-cell RNA sequencing data. **A** (iv), **B** (iv), **C** (iv), **D** (iv), **E** (iv), **F** (iii) and **G** (iv) summarize the *ACE2* expression level in each tissue, where Fetal_SI stands for the supplementary datasets of fetal tissue (refer to Supplementary Table 1), and Fetal_Ori and Adult_Ori stand for the datasets used in the manuscript. **A** (v), **B** (v), **C** (v), **D** (v), **E** (v), **F** (iv) and **G** (v) summarize the *TMPRSS2* expression level in each tissue, where Fetal_SI stands for the supplementary datasets of fetal tissue (refer to Supplementary Table 1), and Fetal_Ori and Adult_Ori stand for the datasets used in the manuscript.
