# Supplementary material for: Single-cell expression profiles of ACE2 and TMPRSS2 reveals potential vertical transmission and fetus infection of SARS-CoV-2
Source: Aging (Albany NY). 2020 Oct 26;12(20):19880–97. doi: 10.18632/aging.104015 (PMC7655214; doi:10.18632/aging.104015)
Supplement: Supplementary Table 1 [file aging-12-104015-s002..pdf]

SUPPLEMENTARY TABLE

Supplementary Table 1. Expression level of *ACE2* in placenta and other fetal organs.

Organs/Tissues	ID	Database
Fetal heart	GSE106118	GEO
Fetal muscle	GSE147457	GEO
Fetal lung	E-MTAB-8821	EMBL-EBI
Placenta	GSE89497	GEO
Fetal kidney	4834STDY7002881	EMBL-EBI
	FCAImmP7292032	EMBL-EBI
Fetal liver	4834STDY7002882	EMBL-EBI
	FCAImmP7198434	EMBL-EBI
